# Development of biodegradable triazine–chitosan nanocomposites enhanced with magnetic and silica nanoparticles for improved drilling fluid properties

**DOI:** 10.1039/d6ra01004k

**Published:** 2026-06-04

**Authors:** Sara Motahari, Ali Reza Kiasat, Arash Mouradzadegun, Abbas Khaksar Manshad, Seifollah Jamalpour

**Affiliations:** a Department of Chemistry, Faculty of Science, Shahid Chamran University of Ahvaz Ahvaz Iran akiasat@scu.ac.ir; b School of Chemistry, College of Science, University of Tehran Tehran 1417614411 Iran Moradzadegun@ut.ac.ir; c Department of Petroleum Engineering, Abadan Faculty of Petroleum Engineering, Petroleum University of Technology (PUT) Abadan Iran; d Department of Chemical Engineering, Shahid Chamran University of Ahvaz Ahvaz Iran

## Abstract

Despite continuous advancements in drilling fluid technology, achieving a balance between operational efficiency, environmental compatibility, and effective filtration control remains a persistent challenge. In this study, two novel chitosan-based nanocomposites—magnetic and silica nanoparticles functionalized with a triazine–chitosan network crosslinked by glutaraldehyde (Fe_3_O_4_@SiO_2_@melamine–chitosan and SiO_2_@melamine–chitosan)—were synthesized and systematically evaluated as additives for water-based drilling fluids. The performance enhancement is attributed to the synergistic interactions between the chitosan backbone, triazine-rich melamine units, and the nanoparticle cores, which collectively promote strong interfacial bonding, improved dispersion stability, and effective fluid–solid interactions. Rheological and filtration evaluations demonstrated a pronounced reduction in both American Petroleum Institute and high-pressure/high-temperature fluid loss with increasing nanocomposite concentration, with SiO_2_@melamine–chitosan exhibiting superior filtration control. At a nanocomposite concentration of 3000 ppm, the standard filtration loss decreased from 13 to 7 mL per 30 min, while the HPHT filtration loss was reduced from 30 to 16 mL per 30 min. These improvements are associated with enhanced wellbore stability and reduced formation damage through the formation of thinner, less permeable filter cakes. The filter cake thickness decreased from 2.5 to 1.5 mm. Moreover, the incorporation of the nanocomposites led to a notable increase in yield point—particularly in systems containing functionalized magnetic nanoparticles—indicating improved suspension capacity and flow control, while plastic viscosity remained largely unaffected. The yield point increased from 16 to 30 lb per 100 ft^2^, while plastic viscosity remained within the range of 16–20 cP. Overall, the combined benefits of enhanced rheological performance, effective filtration regulation, together with the environmentally favorable characteristics associated with chitosan-based materials, underscore the potential of these surface-engineered nanocomposites as potentially environmentally compatible and efficient additives for advanced water-based drilling fluid formulations.

## Abstract 2

Despite continuous advancements in drilling fluid technology, achieving a balance between operational efficiency, environmental compatibility, and effective filtration control remains a persistent challenge.

## Graphical abstract

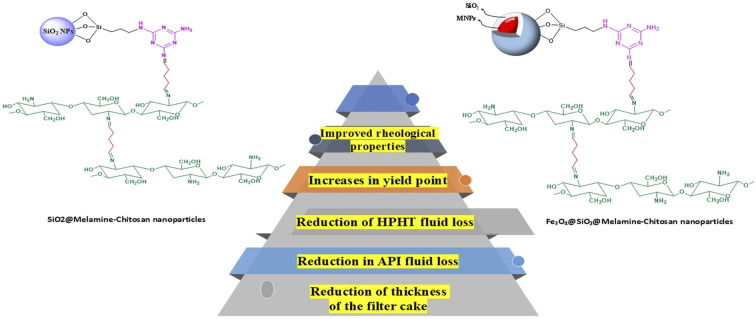

## 1. Introduction

In recent years, the drilling industry has encountered increasing challenges associated with environmental protection, regulatory constraints, and the need to optimize drilling efficiency, which has intensified the demand for high-performance and environmentally benign drilling fluid systems.^1–3^ Shale formations represent some of the most important unconventional hydrocarbon resources worldwide; however, their complex mineralogical composition—typically rich in clay, mud, and silt—poses significant operational challenges during drilling.^4,5^ These formations are highly sensitive to fluid–rock interactions, making drilling fluid design a critical factor in maintaining wellbore stability and minimizing formation damage.^6,7^

Water-based drilling fluids (WBDFs) are widely favored over oil-based systems due to their lower environmental impact, cost effectiveness, and ease of disposal.^1,6^ Nevertheless, WBDFs often exhibit limited performance in reactive shale formations, particularly under high-pressure/high-temperature (HPHT) conditions. Excessive fluid loss, clay hydration and dispersion, and subsequent wellbore instability remain persistent issues.^7,8^ Consequently, the development of multifunctional additives capable of simultaneously enhancing filtration control, rheological stability, and wellbore integrity has become an essential research focus in modern drilling fluid technology.^3,9–12^

Nanocomposites have emerged as promising advanced materials across a wide range of applications, owing to their tunable physicochemical properties and high surface-to-volume ratios.^8,12^ In drilling fluid systems, nanoparticles can effectively reduce fluid loss by plugging micro- and nano-scale pores within the filter cake and near-wellbore formations, thereby limiting filtrate invasion and mitigating formation damage.^8,13,14^ Among the various nanoparticle candidates, iron-based magnetic nanoparticles—particularly Fe_3_O_4_ magnetic nanoparticles (MNPs)—have attracted significant attention due to their chemical stability, relatively low cost, and generally low toxicity.^13,15–17^ In parallel, silica nanoparticles have been widely studied for their ability to improve rheological behavior, enhance thermal stability, and promote the formation of thin, low-permeability filter cakes.^18–23^

Recent studies have demonstrated that both magnetic and silica nanoparticles can substantially improve drilling fluid performance by enhancing viscosity profiles, reducing filtrate loss, and improving mud stability under harsh drilling conditions.^2,12,15,24^ In particular, iron-based nanomaterials have been shown to improve yield stress and suspension capacity, while silica nanoparticles contribute to improved filtration control and thermal resistance.^17,21–25^ An additional advantage of MNPs lies in their magnetic responsiveness, which enables their separation and potential reuse through the application of an external magnetic field, thereby improving economic feasibility and sustainability.^2,15,24,26^ Moreover, MNPs have been reported to reduce wellbore instability by limiting fluid invasion into formations and enhancing adhesion to wellbore walls, leading to improved drilling efficiency and reduced reliance on conventional, often toxic, chemical additives.^2,13,17^

Despite these promising advances, non-functionalized magnetic and silica nanoparticles frequently suffer from aggregation and poor dispersion stability in complex drilling fluid environments, which limits their effectiveness and reproducibility.^8,9^ To address these limitations, recent research has increasingly focused on surface modification strategies, including polymer coating, organic–inorganic hybridization, and functional group anchoring.^26–31^ Techniques such as silane coupling, polymer blending, and nanostructured templating have proven effective in enhancing nanoparticle dispersion, interfacial compatibility, and mechanical stability within drilling fluid systems.^8,15,27,29,30^ In this context, the use of biodegradable and environmentally benign polymers as surface modifiers has gained particular attention as part of broader sustainability initiatives within the oil and gas industry.^8–10,12,15^

Biopolymers such as chitosan, sodium alginate, and β-cyclodextrin have been extensively employed as organic modifiers in heterogeneous catalysis, nanoadsorption, and drug delivery applications, owing to their biodegradability, functional versatility, and favorable interaction characteristics.^31,32^ More recently, these materials have been explored as sustainable additives in WBDFs.^9,10,32,33^ Among them, chitosan-based additives and nanocomposites are especially attractive due to their natural origin, non-toxic nature, and strong electrostatic interactions with negatively charged clay surfaces.^14,34,35^ These interactions can enhance shale inhibition, improve rheological behavior, and promote the formation of compact, low-permeability filter cakes, thereby reducing filtrate loss and helping to maintain wellbore integrity under challenging drilling conditions.^34,36,37^

Notably, Doley *et al.* demonstrated that carboxymethyl chitosan could reduce filtrate loss by up to 53.12% and improve shale recovery by 34.12% compared with conventional inhibitors such as polyethylene glycol, highlighting the potential of chitosan-based materials as effective and environmentally friendly drilling fluid additives.^34^ Despite extensive investigations into magnetic- and silica-nanoparticle-based systems, however, there are currently no reports on the synthesis of magnetic and silica nanoparticles functionalized with a triazine–chitosan network crosslinked by glutaraldehyde, nor on their application in WBDFs. Building on insights from our previous studies and recent advances in surface-engineered nanocomposites, the present work explores the synthesis and application of melamine-modified MNPs incorporated into a glutaraldehyde-crosslinked chitosan matrix for sustainable WBDF formulations.^34,38,39^ The enhanced performance of the proposed nanocomposites is attributed to the synergistic contribution of melamine-derived triazine units and chitosan functional groups.^34,39,40^ The amine-rich triazine structure provides multiple interaction sites, while chitosan contributes hydroxyl and amino functionalities that promote strong interfacial bonding, improved dispersion stability, and effective fluid–solid interactions.^35,39,41,42^ It is anticipated that this integrated organic–inorganic design will contribute to the development of high-performance, biodegradable drilling fluid additives capable of improving drilling efficiency while minimizing environmental impact.^34,35^ The primary objective of this research is to evaluate and demonstrate the effects of these nanocomposites on the rheological properties and filtration loss characteristics of drilling fluids, with a particular focus on their ability to achieve superior wellbore stability and reduced formation damage while maintaining environmental sustainability. Evaluation of the interaction of the synthesized nanoparticles within the drilling fluid matrix, we aim to provide innovative solutions that meet the growing demand for efficient and eco-friendly drilling technologies.

## 2. Experimental

### 2.1. Instruments and materials

All chemicals and reagents were obtained from reputable commercial suppliers (Merck, Fluka, Aldrich, and Acros) and were used as received without further purification. Melamine (MA, 99%, Merck), FeCl_2_·4H_2_O (99%, Merck), FeCl_3_·6H_2_O (99%, Merck), glutaraldehyde (25% aqueous solution, Aldrich), methanol (98%, Merck), and tetraethyl orthosilicate (TEOS, 99%, Merck), and chloropropyltrimethoxysilane (CPTMS, 98%), tetraethyl orthosilicate (TEOS, 97%, Merck) were used to synthesis SiO_2_@melamine–chitosan and Fe_3_O_4_@SiO_2_@melamine–chitosan nanocomposite. Drilling fluid materials, bentonite (montmorillonite clay, Sigma-Aldrich) and barite (barium sulfate, Sigma-Aldrich, B3758, assay 97.5–100.5%) were used as received for preparation of the water-based drilling fluids. Potassium chloride (KCl, Sigma-Aldrich, P3911, ACS Reagent, 99.0–100.5%) was used to prepare the saline brine. Deionized water was used throughout. Because bentonite is a naturally occurring clay mineral, it is typically specified by mineral identity/grade and physical properties rather than a single assay (purity) value; therefore, supplier and material identity are reported.

The melting points of the synthesized compounds were determined using a digital electrothermal apparatus (Electrothermal 9200, England) with open capillary tubes and are reported uncorrected. Fourier-transform infrared (FT-IR) spectra were recorded in the 400–4000 cm^−1^ range using spectroscopic-grade KBr pellets on a Galaxy Series FT-IR 5000 spectrometer (Thermo Fisher Scientific, USA, Madison). Thermal stability was evaluated *via* thermogravimetric analysis (TGA) on a Mettler TA4000 system (Mettler Toledo, Switzerland, Greifensee) under controlled heating conditions. Morphological and structural features were examined by field-emission scanning electron microscopy (FE-SEM) using a Zeiss Sigma microscope (Carl Zeiss AG, Germany, Oberkochen) equipped with energy-dispersive X-ray spectroscopy (EDS) for elemental mapping (Oxford Instruments, UK, Abingdon). All samples were coated with a thin layer of gold to enhance conductivity and improve imaging quality. The sputtering was performed using a Quorum Q150R Sputter (Quorum Technologies, UK, Laughton) under these conditions (sputtering time: 30 seconds, current: 20 mA, pressure: 0.1 mbar, coating thickness: approximately 10–20 nm). The specific surface area and pore size distribution of the samples were determined using the Brunauer–Emmett–Teller (BET) and Barrett–Joyner–Halenda (BJH) methods, respectively, on a BELSORP Mini analyzer (MicrotracBEL Corp., Japan). The hydrodynamic particle size and zeta potential were also evaluated *via* dynamic light scattering (DLS) using an SZ-100 analyzer (Horiba, Japan). Crystallographic properties were investigated by X-ray diffraction (XRD) using a Holland Philips X'Pert X-ray diffractometer (Malvern Panalytical, Netherlands, Almelo) with Cu Kα radiation (*λ* = 0.15405 nm) over a 2*θ* range of 20°–80°.

### 2.2. Preparation of Fe_3_O_4_ nanoparticles

Magnetite nanoparticles (Fe_3_O_4_ MNPs) were synthesized *via* a modified co-precipitation method. In a typical procedure, 2.70 g (10 mmol) of FeCl_3_·6H_2_O and 0.99 g (5 mmol) of FeCl_2_·4H_2_O were dissolved in 60 mL of double-distilled water under a nitrogen atmosphere. The solution was stirred vigorously at 1200 rpm until the salts were fully dissolved. The temperature was then increased to 60 °C and maintained for 25 minutes with continuous stirring. Following this, 10 mL of 25% aqueous ammonia was added dropwise while keeping the temperature constant at 60 °C under a nitrogen atmosphere. The reaction mixture was stirred for an additional 2 hours to ensure complete precipitation. The resulting black Fe_3_O_4_ nanoparticles were isolated using an external magnet, washed thoroughly three times with distilled water and then with ethanol, and finally dried under vacuum at 50 °C to obtain the pure magnetite nanoparticles.^39^

### 2.3. Synthesis of Fe_3_O_4_@SiO_2_ nanoparticles

Silica-coated magnetite nanoparticles (Fe_3_O_4_@SiO_2_) were synthesized *via* the Stöber method, a well-established approach for forming uniform silica shells on magnetic cores. In a typical procedure, 1 g of Fe_3_O_4_ nanoparticles was ultrasonically dispersed for 15 minutes in a mixture of 80 mL ethanol, 20 mL deionized water, and 3 mL of 25% ammonium hydroxide (NH_4_OH) to obtain a homogeneous suspension. Subsequently, 1.5 mL of tetraethyl orthosilicate (TEOS) was added dropwise under continuous stirring. The reaction mixture was then stirred mechanically at 60 °C for 12 hours under a nitrogen atmosphere to facilitate silica coating. The resulting Fe_3_O_4_@SiO_2_ core–shell nanoparticles were separated magnetically, washed thoroughly several times with deionized water and ethanol to remove residual reactants, and dried in an oven at 50 °C for 24 hours to yield the final product.^29,40^

### 2.4. Synthesis of Fe_3_O_4_@SiO_2_@CPTMS nanoparticles

Chloro-functionalized MNPs (Fe_3_O_4_@SiO_2_@CPTMS) were synthesized *via* a modified procedure adapted from a previously reported method. In a typical synthesis, 2 g of silica-coated Fe_3_O_4_ nanoparticles was dispersed in 60 mL of toluene, followed by the addition of 2 mL of 3-chloropropyltriethoxysilane (CPTMS). The reaction mixture was stirred under reflux in a nitrogen atmosphere for 24 hours. The resulting Fe_3_O_4_@SiO_2_@CPTMS nanoparticles were separated magnetically, washed thoroughly four times with water (60 mL each), and dried under vacuum at 50 °C to yield the final chloro-functionalized product.^29,40^

### 2.5. Preparation of Fe_3_O_4_@SiO_2_@melamine nanoparticles

Fe_3_O_4_@SiO_2_@CPTMS nanoparticles (1.0 g) were dispersed in 30 mL of dry methanol and ultrasonicated for 30 minutes to obtain a homogeneous suspension. Subsequently, 2,4,6-triamino-1,3,5-triazine (melamine) (0.35 g, 3 mmol) was added to the reaction mixture. The mixture was then heated under reflux in a nitrogen atmosphere and stirred continuously for 24 hours to facilitate surface modification. Upon completion, the resulting Fe_3_O_4_@SiO_2_@melamine nanoparticles were separated magnetically, washed thoroughly several times with hot ethanol to remove residual and unreacted melamine, and dried under vacuum at 50 °C for 8 hours to yield the desired melamine-functionalized nanomaterial.

### 2.6. Preparation of Fe_3_O_4_@SiO_2_@melamine–chitosan nanoparticles

**Scheme 1:**
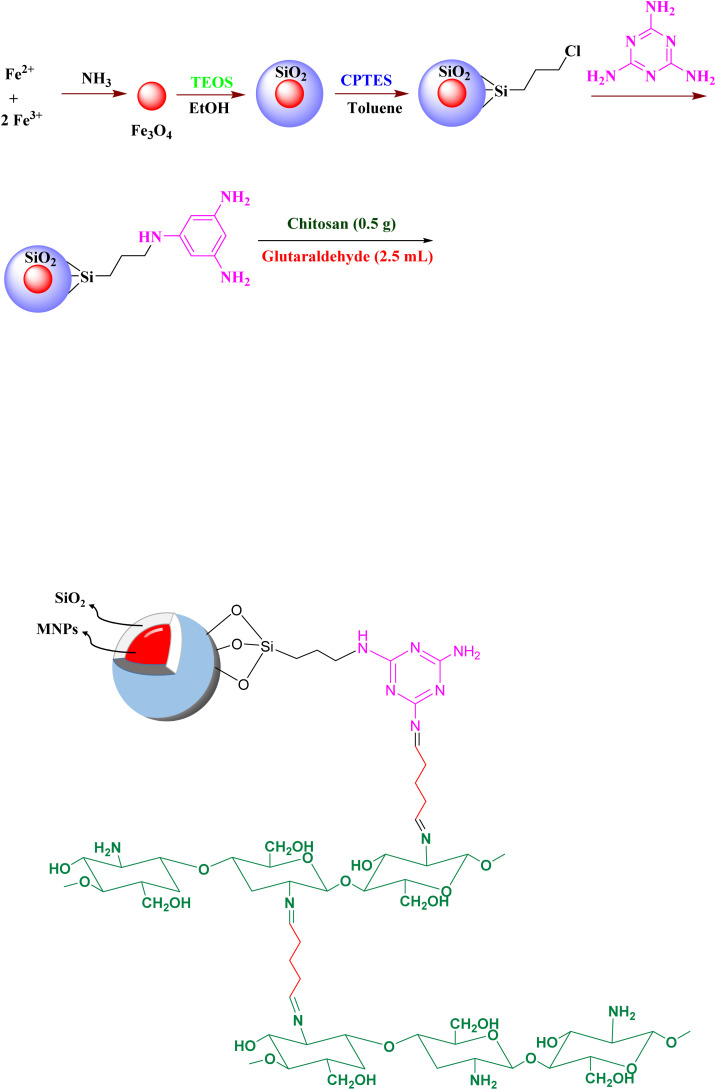
Synthesis of Fe_3_O_4_@SiO_2_@melamine–chitosan nanoparticles.

To synthesize chitosan-modified Fe_3_O_4_@SiO_2_@melamine–chitosan nanoparticles, 0.5 g of chitosan was dissolved in 50 mL of a 2% acetic acid solution and stirred at 60 °C for 2 hours. Subsequently, 0.5 g of Fe_3_O_4_@SiO_2_@melamine nanoparticles was added to the chitosan solution and dispersed *via* ultrasonication for 30 minutes. Finally, 2.5 mL of 25% glutaraldehyde was added, and the pH of the mixture was adjusted to 10 using a 1 M NaOH solution. The resulting gel was freeze-dried (Scheme 1).

### 2.7. Synthesis of SiO_2_@melamine–chitosan nanocomposite

**Scheme 2:**
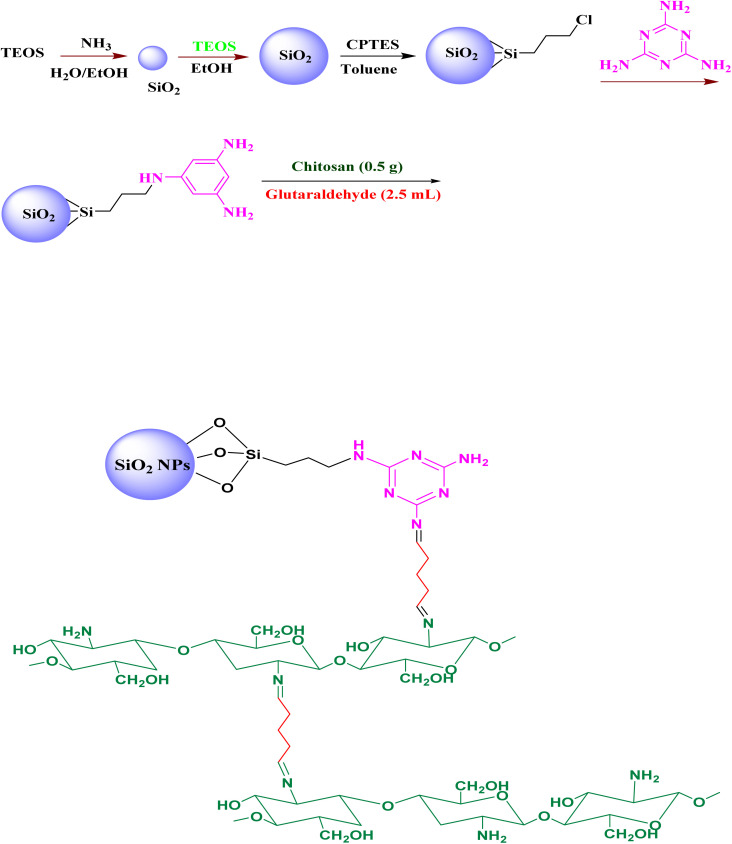
Synthesis of SiO_2_@melamine–chitosan nanoparticles.

In this procedure, 2.2 mL of TEOS was dissolved in 100 mL of ethanol.^42^ A solution containing 1.5 mL of NH_4_OH and 1.8 mL of water was then added slowly to the TEOS solution. The mixture was stirred at room temperature for 24 hours. The resulting product was centrifuged and washed with water. Finally, the SiO_2_ nanoparticles were dried under reduced pressure. The subsequent modification steps for the SiO_2_ NPs followed the same procedure used to synthesize the Fe_3_O_4_@SiO_2_@melamine–chitosan nanoparticles (Scheme 2).

### 2.8. Preparation of nanocomposite dispersions

Nanocomposite dispersions were prepared at concentrations of 0 (control), 1000, 2000, and 3000 ppm for a total volume of 350 mL. Based on the definition of ppm (mg L^−1^), the required amounts of nanocomposite were accurately weighed as 0.35, 0.70, and 1.05 g for 1000, 2000, and 3000 ppm, respectively. Each weighed nanocomposite sample was gradually added to the base liquid (deionized water for fresh systems or KCl brine for saline systems) under continuous stirring. The resulting suspension was probe-ultrasonicated for 10 min to ensure uniform dispersion and minimize particle agglomeration. The freshly prepared nanocomposite dispersion was directly used for drilling mud preparation without storage.

### 2.9. Preparation of fresh and KCl-based water-based drilling fluids

Fresh and KCl-based water-based drilling fluids were prepared in both light (9.1 ppg) and heavy (12 ppg) forms following the same general procedure. For KCl-based systems, potassium chloride (10.5 g) was first dissolved in 350 mL deionized water to obtain a concentration of 3 wt%. The required amount of nanocomposite dispersion was then added and probe-ultrasonicated for 10 min. Bentonite (22.5 g) was gradually introduced under continuous stirring to prevent agglomeration and ensure proper hydration. The mixture was stirred using a high-speed laboratory mixer at approximately 10 000 rpm for 20 min until a homogeneous mud was obtained. The prepared light mud samples were allowed to age for 16 h at ambient temperature. Heavyweight drilling fluids (12 ppg) were prepared by weighting the aged light mud formulations with barite. Barite was gradually added under continuous stirring until the target density was achieved (185.08 g per 350 mL). The mixture was stirred for an additional 15 min to ensure uniformity. The detailed weight compositions of fresh and KCl-based drilling fluids (light and heavy) at different nanocomposite concentrations are provided in Tables 1–4.

**Table 1:** Fresh water-based drilling fluids – light mud (9.1 ppg)

Nanocomposite (ppm)	Water (%)	Bentonite (%)	Nanocomposite (%)
0	93.96	6.04	—
1000	93.88	6.03	0.09
2000	93.78	6.03	0.19
3000	93.70	6.02	0.28

**Table 2:** Fresh water-based drilling fluids – heavy mud (12 ppg)

Nanocomposite (ppm)	Water (%)	Bentonite (%)	Barite (%)	Nanocomposite (%)
0	62.77	4.04	33.19	—
1000	62.73	4.03	33.17	0.06
2000	62.69	4.03	33.15	0.13
3000	62.65	4.03	33.13	0.19

**Table 3:** KCl-based drilling fluids – light mud (9.1 ppg)

Nanocomposite (ppm)	Water (%)	KCl (%)	Bentonite (%)	Nanocomposite (%)
0	94.14	3.00	2.86	—
1000	94.05	3.00	2.85	0.10
2000	93.95	3.00	2.85	0.20
3000	93.86	3.00	2.84	0.30

**Table 4:** KCl-based drilling fluids – heavy mud (12 ppg)

Nanocomposite (ppm)	Water (%)	KCl (%)	Bentonite (%)	Barite (%)	Nanocomposite (%)
0	61.58	1.90	3.96	32.56	—
1000	61.54	1.90	3.96	32.54	0.06
2000	61.50	1.90	3.95	32.52	0.12
3000	61.46	1.90	3.95	32.50	0.18

### 2.10. Measurement of mud weight using a mud balance

The density of the mud samples was measured using a mud balance according to API RP 13B-1. The device was calibrated and leveled prior to measurement. The balance cup was filled with the mud sample, excess fluid was removed from the overflow port to ensure a constant volume, and the movable weight was adjusted along the graduated arm until equilibrium was reached. The mud density was read directly and recorded in pounds per gallon (ppg) at ambient temperature.

### 2.11. Measurement of rheological properties/viscosity using a rotational viscometer

To evaluate rheological properties, the mud sample was poured into the cup of the rotational viscometer according to API RP 13B-1. Once the rotor was set to the standard position, readings were taken at standard speeds (600 and 300 rpm) all measurements were performed at ambient temperature. Apparent viscosity was calculated as half the reading at 600 rpm, plastic viscosity as the difference between the readings at 600 and 300 rpm, and yield point as the difference between the reading at 300 rpm and the plastic viscosity. Additionally, gel strengths were measured after static aging periods of 10 s and 10 min.

### 2.12. Measurement of API filtration loss (LPLT) using an API filter press

The API filtration loss was determined using a standard API filter press according to API RP 13B-1. The apparatus was assembled with a standard filter paper in place. The cell was then filled with the mud sample, and a standard pressure of 100 psi was applied. The volume of filtrate collected over the 30-minute test period was recorded in milliliters. Upon completion, the filter cake was carefully separated, and its thickness was measured. All measurements were conducted at ambient temperature.

### 2.13. Measurement of HPHT filtration loss using an HPHT filter press

HPHT filtration tests were conducted using an HPHT filter press according to API RP 13B-1. The device was assembled and sealed with the appropriate filter medium in place. The filtration cell was filled with the mud sample and securely closed. After reaching the target test temperature of 150 °C, a differential pressure of 500 psi was applied. The filtrate volume collected over the standard 30 min test period was recorded. Upon completion of the test, the filter cake was removed, and its thickness was measured.

All experimental measurements were conducted in triplicate, and the reported values correspond to the average of repeated tests.

## 3. Results and discussion

### 3.1. Synthesis and characterization

In this work, a novel magnetically separable nanocomposite (Fe_3_O_4_@SiO_2_@melamine–chitosan) and its silica-based analogue (SiO_2_@melamine–chitosan) were successfully synthesized through a multi-step functionalization process (Schemes 1 and 2). The synthesis procedure of magnetite (Fe_3_O_4_) nanoparticles is the chemical co-precipitation of Fe(iii) and Fe(ii) salts in an ammonia solution. These Fe_3_O_4_ nanoparticles were then coated with a silica layer using the Stöber method to form Fe_3_O_4_@SiO_2_. Surface modification of the silica shell with CPTMS yielded chloro-functionalized Fe_3_O_4_@SiO_2_–Cl nanoparticles. These subsequently reacted with melamine (2,4,6-triamino-1,3,5-triazine) to form Fe_3_O_4_@SiO_2_@melamine–chitosan. In the final step, chitosan was covalently anchored to this triazine-functionalized surface *via* crosslinking with glutaraldehyde, resulting in the target Fe_3_O_4_@SiO_2_@melamine–chitosan nanocomposite. Comprehensive characterization confirmed the structural integrity and successful surface modification at each stage. The collective results verify the effective stepwise synthesis, demonstrating the material's potential as a biodegradable additive for drilling fluids.

**Fig. 1:**
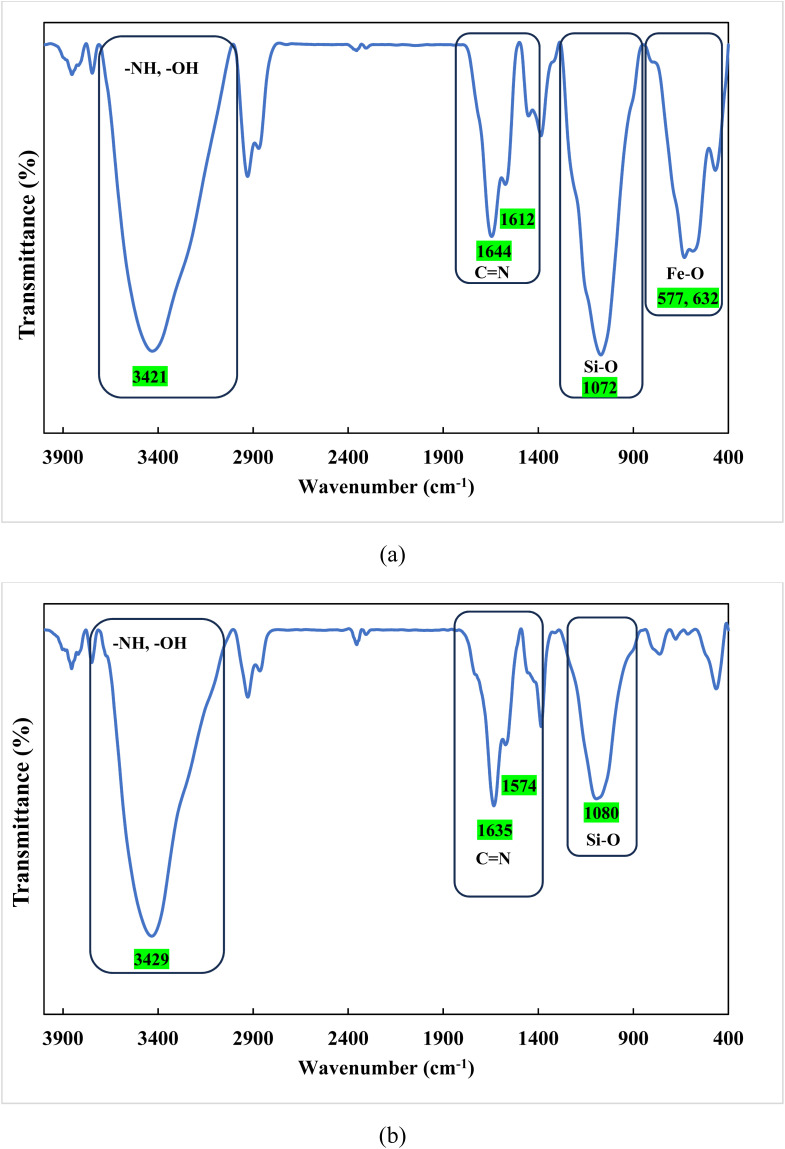
The FT-IR spectra of Fe_3_O_4_@SiO_2_@melamine–chitosan (a) and SiO_2_@melamine–chitosan (b).

FTIR spectroscopy was used to monitor the progressive structural modifications during the synthesis of the Fe_3_O_4_@SiO_2_@melamine–chitosan and SiO_2_@melamine–chitosan nanocomposites (Fig. 1a and b). The FTIR spectrum of Fe_3_O_4_@SiO_2_@melamine–chitosan (Fig. 1a) revealed a strong absorption peak at 578 cm^−1^, corresponding to the characteristic Fe–O stretching vibration of the magnetite core.^43^ A broad band around 3427 cm^−1^ was attributed to O–H stretching from surface hydroxyl groups, adsorbed water and N–H.^43–45^ The presence of the silica shell was confirmed by absorption bands at approximately 470, 912, and 1071 cm^−1^, representing Si–O bond vibrations (rocking, bending, and stretching modes).^43^ Modification with CPTMS to synthesize Fe_3_O_4_@SiO_2_–Cl introduced peaks at 2931 cm^−1^ and 2867 cm^−1^, corresponding to asymmetric and symmetric –CH_2_– stretching vibrations.^45^ Subsequent functionalization with melamine yielded characteristic bands for the triazine ring, most notably a sharp peak at 1647 cm^−1^ attributed to C=N stretching. The final step involving chitosan and glutaraldehyde crosslinking produced a new absorption peak at 1722 cm^−1^, indicating the formation of imine bonds (C=N) from the reaction between glutaraldehyde and the amino groups of melamine and chitosan, confirming the successful synthesis of the target nanocomposite. The FTIR spectrum of the SiO_2_@melamine–chitosan nanocomposite (Fig. 1b) showed characteristic bands: a broad region from 3000 to 3422 cm^−1^ (O–H and N–H stretching), peaks at 1741 cm^−1^ and 1635 cm^−1^ (C=N stretching from melamine and imines), and a strong band at 1083 cm^−1^ (Si–O–Si stretching). These features confirm the successful grafting of the melamine–chitosan organic network onto the silica nanoparticle surface. Collectively, the FTIR results verify the stepwise functionalization and successful synthesis of both nanocomposite structures.

**Fig. 2:**
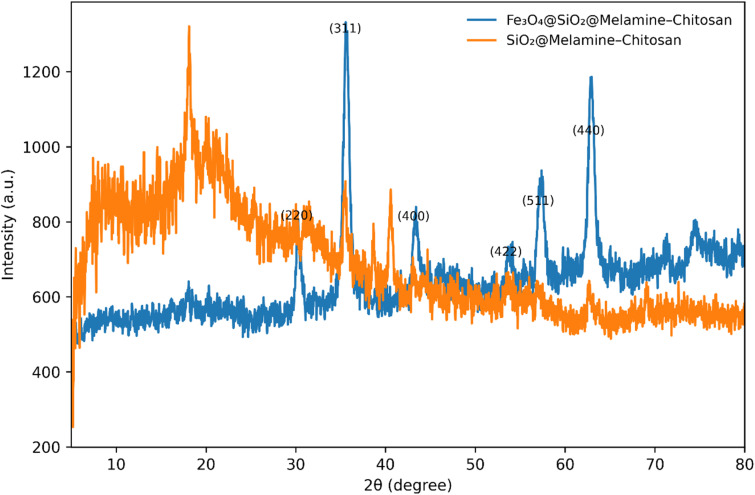
XRD pattern of Fe_3_O_4_@SiO_2_@melamine–chitosan and SiO_2_@melamine–chitosan.

The crystalline structures of Fe_3_O_4_@SiO_2_@melamine–chitosan and SiO_2_@melamine–chitosan nanocomposites were examined by XRD (Fig. 2). For direct comparison, the two patterns are presented together in a single merged figure, and the assignment of panels was carefully verified. The Fe_3_O_4_-containing nanocomposite exhibits the characteristic reflections of cubic spinel magnetite at 2*θ* ≈ 30.1°, 35.5°, 43.1°, 53.5°, 57.0° and 62.6°, which can be indexed to the (220), (311), (400), (422), (511) and (440) planes, respectively, confirming that the crystalline Fe_3_O_4_ core remains preserved after coating and functionalization.^46^ A broad diffuse halo centered around 2*θ* ≈ 20–25° is attributed to the amorphous SiO_2_ shell and the organic functional layers.^47^ In contrast, the SiO_2_@melamine–chitosan sample is dominated by a broad diffuse halo centered at 2*θ* ≈ 20–25°, characteristic of amorphous silica and short-range Si–O–Si structural correlations, and shows a weak shoulder around 30–35° with low-intensity undulations extending up to 65°. These secondary features are attributed to partial structural ordering within the melamine–chitosan coating and the underlying SiO_2_ matrix rather than crystalline phases. The pattern does not show any magnetite-type reflections, consistent with its non-magnetic silica-based and predominantly amorphous structure.

**Fig. 3:**
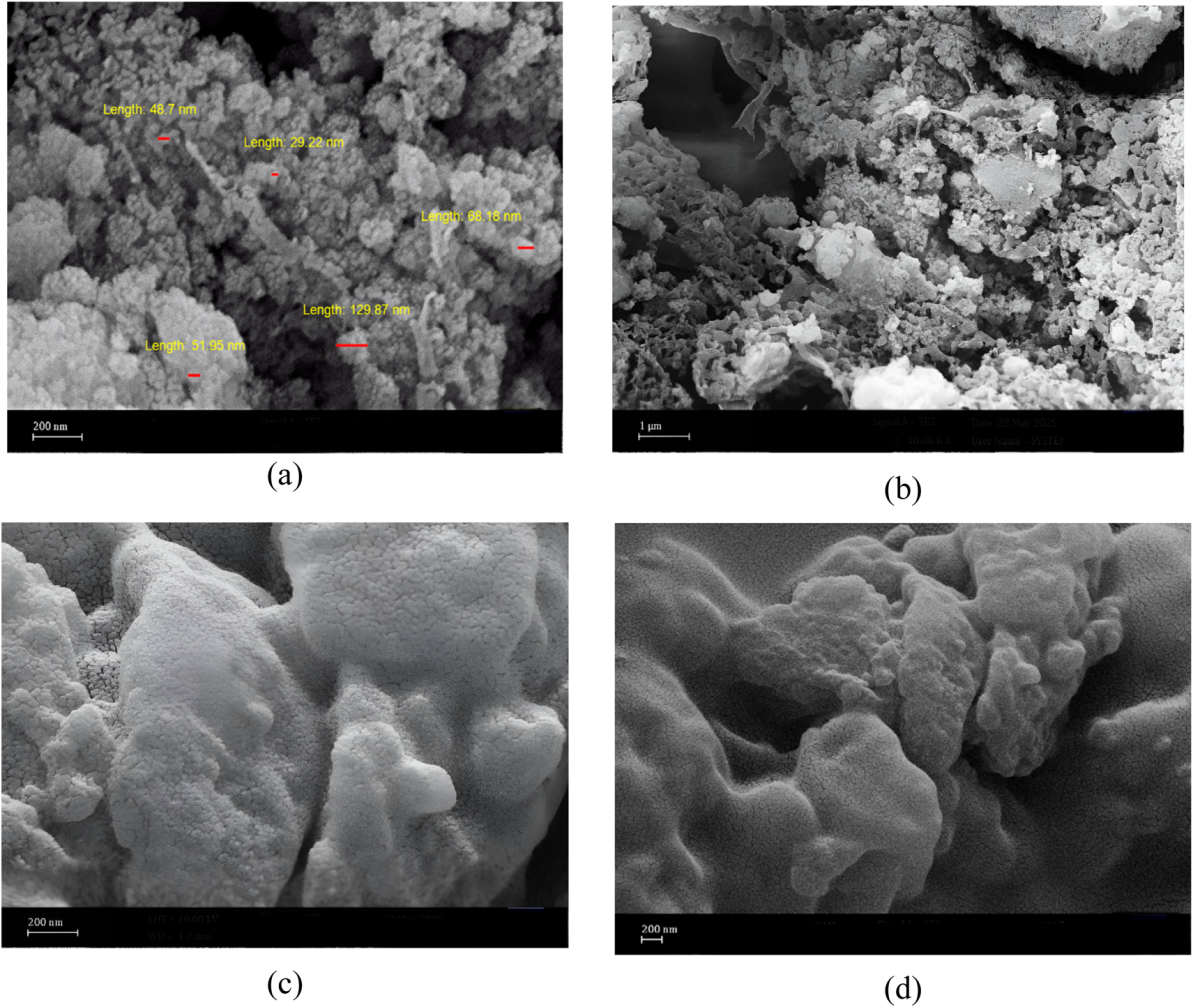
The FE-SEM images of Fe_3_O_4_@SiO_2_@melamine–chitosan (a and b) and SiO_2_@melamine–chitosan nanocomposites (c and d).

FESEM micrographs of the synthesized Fe_3_O_4_@SiO_2_@melamine–chitosan (Fig. 3a and b) and SiO_2_@melamine–chitosan (Fig. 3c and d) illustrate their heterogeneous nature, surface morphology, and structural integrity. In the magnetic nanocomposite (Fig. 3a and b), the images reveal a heterogeneous surface composed of spherical particles, indicating crystalline domains. The particles are generally well-dispersed with minimal aggregation, though some stacking is observed, likely due to magnetic interactions. In the silica-based nanocomposite (Fig. 3c and d), the FESEM images show that the spherical morphology of the silica nanoparticles is retained. The surface appears rough and heterogeneous, with particle aggregates that suggest crystalline formations from the grafted organic components.

The differences in particle morphology and aggregation behavior between the two nanocomposites are also noticeable in the FESEM images. The incorporation of magnetite introduces magnetic nanoparticles within the hybrid structure, which can influence the spatial arrangement of the particles. Consequently, the Fe_3_O_4_-based nanocomposite appears to exhibit a relatively more uniform distribution of particles, although some localized stacking can still be observed, likely due to magnetic interactions between particles. In contrast, the SiO_2_-based nanocomposite shows a greater tendency toward aggregation, leading to the formation of relatively denser particle clusters. These observations suggest that the nature of the core material (silica *vs.* magnetite) can affect particle distribution and aggregation behavior, which may influence the overall structural characteristics of the resulting nanocomposites.

**Fig. 4:**
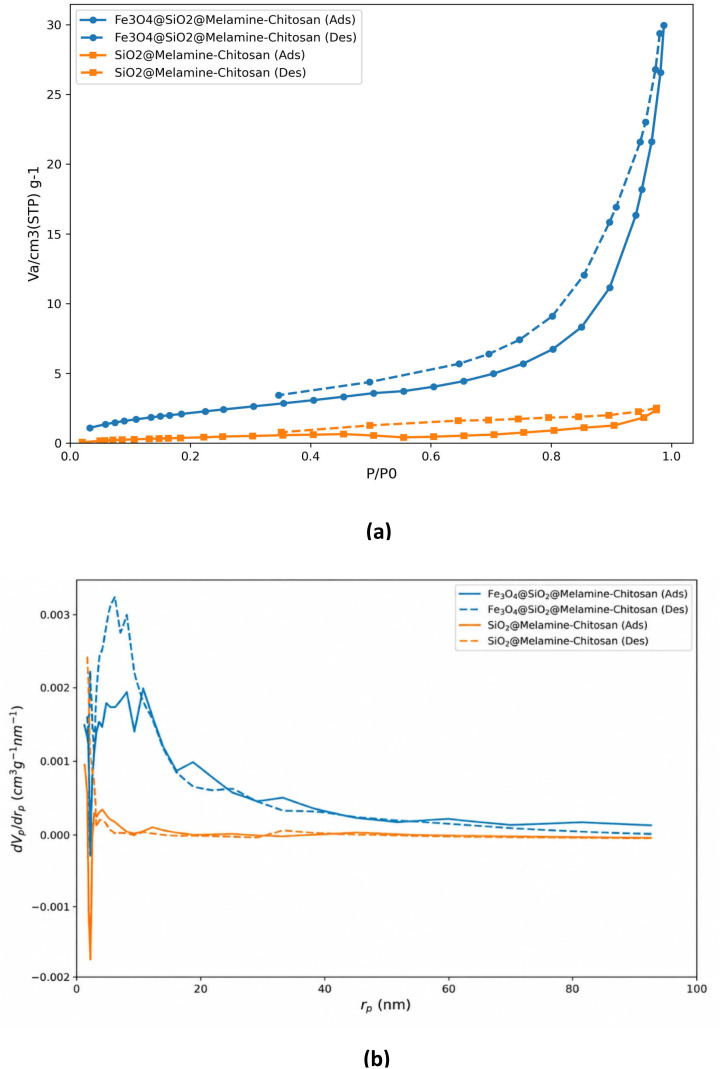
(a) N_2_ adsorption–desorption isotherms and (b) BJH pore size distribution of Fe_3_O_4_@SiO_2_@melamine–chitosan and SiO_2_@melamine–chitosan samples.

The textural properties of the synthesized nanocomposites were evaluated using nitrogen adsorption–desorption analysis. The isotherms (Fig. 4a) exhibit type IV behavior with H3-type hysteresis loops, confirming the mesoporous nature of both materials.

The calculated BET surface area, total pore volume, and pore size distribution parameters are summarized in Table 5. As clearly observed, the magnetic Fe_3_O_4_@SiO_2_@melamine–chitosan composite exhibits substantially higher surface area and pore volume compared to the non-magnetic SiO_2_@melamine–chitosan sample.

**Table 5:** BET surface area and pore characteristics of Fe_3_O_4_@SiO_2_@melamine–chitosan and SiO_2_@melamine–chitosan samples

Sample	*S*_BET_ (m^2^ g^−1^)	Total pore volume (cm^3^ g^−1^)	Average pore diameter (nm)	BJH peak pore size (nm)
Fe_3_O_4_@SiO_2_@melamine–chitosan	8.71	0.0507	23.3	10.7
SiO_2_@melamine–chitosan	1.88	0.0063	13.5	1.22

The BJH pore size distribution (Fig. 4b) further demonstrates a broader mesopore distribution for the magnetic nanocomposite, whereas the non-magnetic counterpart presents a narrower and less developed pore structure. These results are fully consistent with the FESEM observations, indicating that the incorporation of the Fe_3_O_4_ core promotes the formation of a more accessible and heterogeneous porous framework.

The hydrodynamic diameter of the synthesized nanoparticles was investigated using Dynamic Light Scattering (DLS), as summarized in Table 6. The bare Fe_3_O_4_ nanoparticles exhibited a *Z*-average of 300.4 nm. Upon the formation of the silica shell, the main peak size increased to 384.7 nm, confirming the successful coating of the magnetic core. For the final functionalized Fe_3_O_4_@SiO_2_@CPTMS, the *Z*-average reached 720.7 nm. While a high *Z*-average (2843.1 nm) was recorded for the Fe_3_O_4_@SiO_2_ sample due to partial agglomeration in the aqueous medium, the main peak value (384.7 nm) provides a more accurate representation of the individual particle sizes. These DLS results are in good agreement with the FESEM observations, which confirmed the successful synthesis and size evolution of the core–shell structure.

**Table 6:** DLS analysis results for Fe_3_O_4_, Fe_3_O_4_@SiO_2_, and Fe_3_O_4_@SiO_2_@CPTMS nanoparticles

Sample	*Z*-Average (nm)	Main peak/mean (nm)
Fe_3_O_4_	300.4	327.0
Fe_3_O_4_@SiO_2_	2843.1 (agglomerated)	384.7
Fe_3_O_4_@SiO_2_@CPTMS	720.7	190.2

**Fig. 5:**
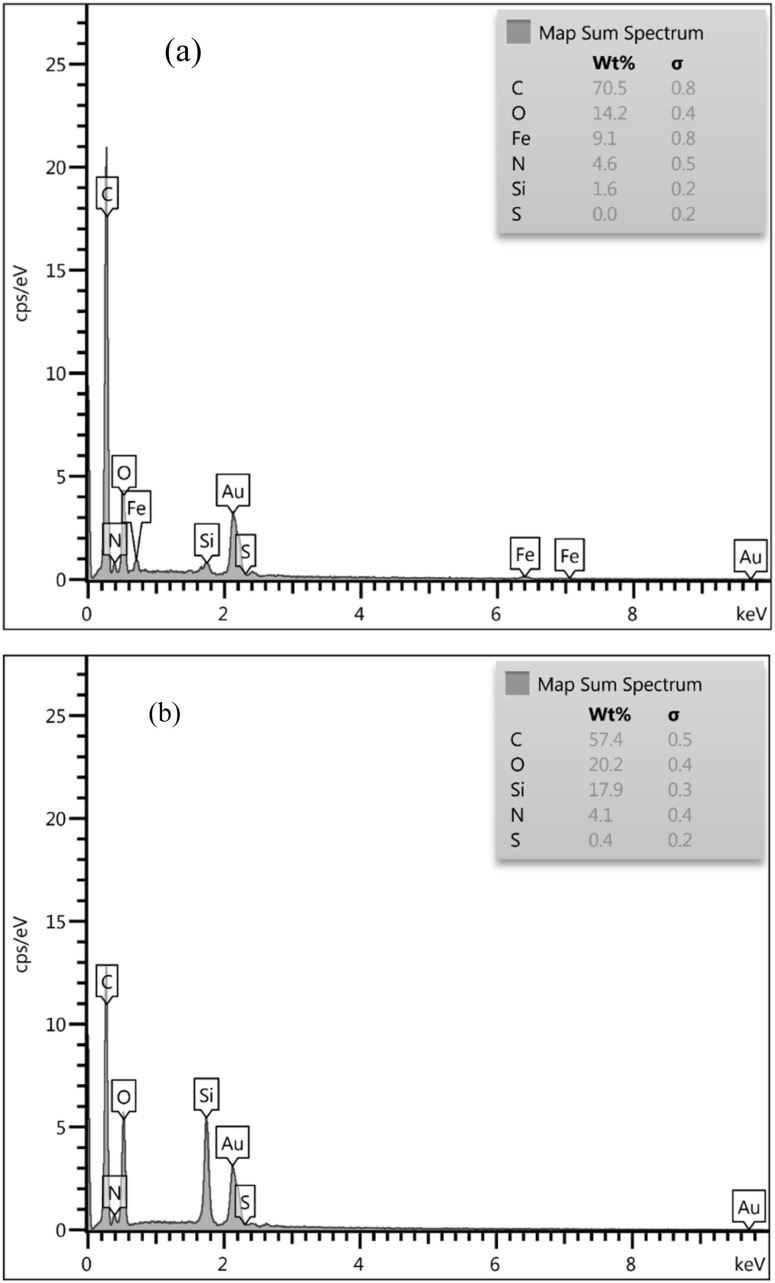
EDX spectrum of (a) Fe₃O₄@SiO₂@melamine–chitosan and (b) SiO_2_@melamine–chitosan.

The successful fabrication and elemental composition of the Fe_3_O_4_@SiO_2_@melamine–chitosan and SiO_2_@melamine–chitosan nanocomposites were confirmed by energy-dispersive X-ray (EDX) analysis (Fig. 5). The EDX spectrum of Fe_3_O_4_@SiO_2_@melamine–chitosan (Fig. 5a) confirmed the presence of Fe, Si, O, N, and C, verifying the synthesis of the modified MNPs. Quantitative analysis revealed the following atomic percentages: carbon (70.5%), oxygen (14.2%), nitrogen (4.6%), iron (9.1%), and silicon (1.6%). The high carbon and detectable nitrogen content directly correspond to the organic melamine and chitosan moieties.

Similarly, the EDX spectrum of SiO_2_@melamine–chitosan (Fig. 5b) confirmed the presence of Si, O, N, and C, indicating the successful grafting of the organic network onto the silica nanoparticles. Quantitative analysis yielded the composition: carbon (57.4%), oxygen (20.2%), silicon (17.9%), and nitrogen (4.1%).

**Fig. 6:**
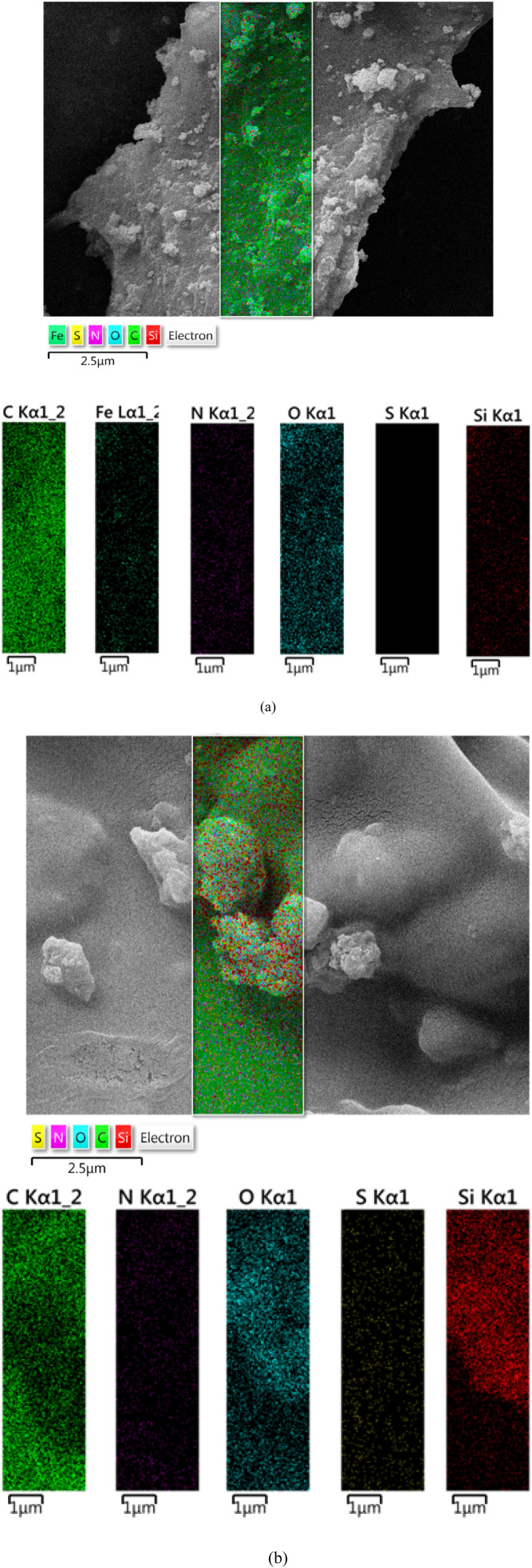
Elemental mapping analysis of Fe_3_O_4_@SiO_2_@melamine–chitosan (a) and SiO_2_@melamine–chitosan nanocomposites (b).

Furthermore, elemental mapping confirmed the uniform distribution of all constituent elements throughout the nanocomposite structures with minimal aggregation. For both Fe_3_O_4_@SiO_2_@melamine–chitosan (Fig. 6a) and SiO_2_@melamine–chitosan (Fig. 6b), the elemental mapping images show that carbon and nitrogen species are well-dispersed and anchored on the nanoparticle surfaces. The similar distribution patterns of carbon and nitrogen suggest a close association, likely resulting from their chemical bonding within the grafted melamine and chitosan network. This uniform elemental dispersion confirms the successful and homogeneous surface functionalization.

**Fig. 7:**
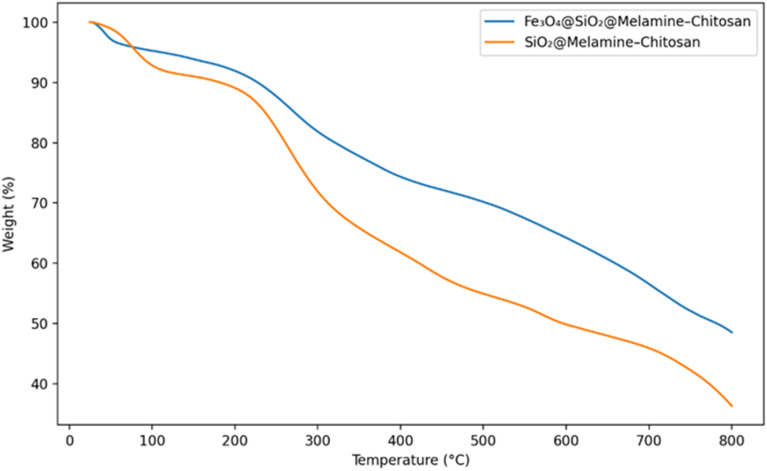
TGA curves of Fe_3_O_4_@SiO_2_@melamine–chitosan and SiO_2_@melamine–chitosan nanocomposites.

Thermogravimetric analysis (TGA) of Fe_3_O_4_@SiO_2_@melamine–chitosan and SiO_2_@melamine–chitosan (Fig. 7) shows a small initial mass loss (∼7% and ∼5%, respectively) in the range of 50–220 °C, which is attributed to the removal of physically adsorbed moisture and residual solvent. For Fe_3_O_4_@SiO_2_@melamine–chitosan, the subsequent mass loss occurs mainly over two overlapping steps: (i) 220–350 °C, assigned to the decomposition of labile organic moieties, and (ii) 350–765 °C, corresponding to further decomposition of the grafted organic framework (chitosan/melamine and crosslinker-related groups).

For SiO_2_@melamine–chitosan, the major weight loss starts at ∼313 °C and extends up to ∼746 °C, consistent with thermal decomposition of the grafted organic components. Overall, the TGA profiles confirm successful functionalization of the nanoparticle surfaces and indicate that both nanocomposites retain a substantial inorganic residue at high temperature, consistent with the presence of SiO_2_ (and Fe_3_O_4_ in the magnetic composite).

**Fig. 8:**
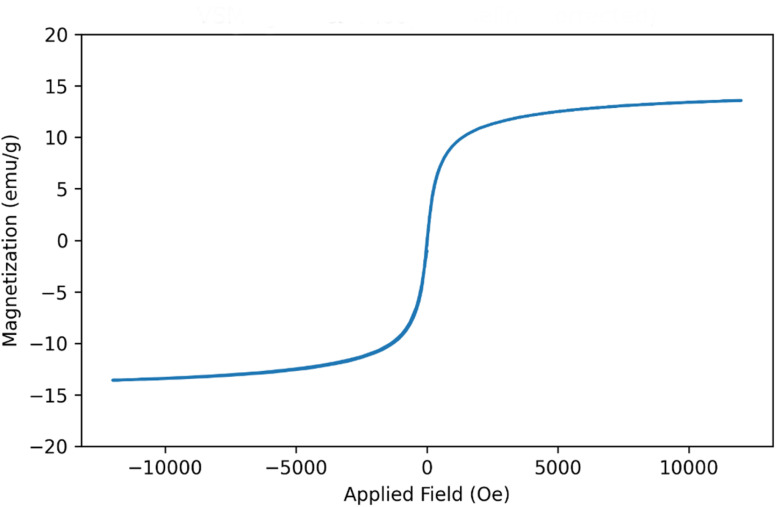
VSM analysis of Fe_3_O_4_@SiO_2_@melamine–chitosan.

Vibrating sample magnetometry of Fe_3_O_4_@SiO_2_@melamine–chitosan (Fig. 8) presents the magnetic hysteresis curve of the sample recorded from −12 000 to +12 000 Oe. The curve shows an S-shaped response with an almost closed loop around the origin, indicating negligible remanence and coercivity. From the baseline-corrected data, the saturation magnetization is *M*_s_ = 13.49 emu g^−1^. The remanent magnetization is *M*_r_ = 0.004 emu g^−1^ (at *H* = 0 Oe), and the coercive field is *H*_c_ ≈ 0.16 Oe, giving a very low squareness ratio *M*_r_/*M*_s_ ≈ 3.12 × 10^−4^. These values are characteristic of soft-magnetic/superparamagnetic nanoparticles at room temperature. The relatively lower *M*_s_ compared with bulk Fe_3_O_4_ can be attributed to the presence of non-magnetic components (*e.g.*, silica/organic coatings) and surface spin-disorder effects; however, the remaining magnetization is sufficient to maintain a clear magnetic response and enable magnetic separation under an external field.

### 3.2. The rheological properties and filtration loss characteristics of fresh mud base (9.1 ppg)

Table 7 demonstrates that both SiO_2_@melamine–chitosan and Fe_3_O_4_@SiO_2_@melamine–chitosan improve the performance of fresh water-based light mud (9.1 ppg), with stronger effects at higher nanoparticle concentrations. Relative to the control (*θ*_600_/*θ*_300_ = 34/22), *θ* readings increase with concentration and reach 44/32 for the silica-based additive at 3000 ppm, while the magnetic nanocomposite shows a larger rise in *θ* readings at comparable loadings. The increase in yield point (from 10 up to 20 lb per 100 ft^2^ for the silica-based series) indicates enhanced low-shear structure and improved suspension capability, which can be attributed to stronger particle association *via* adsorption/hydrogen-bonding interactions enabled by melamine/chitosan functional groups. Meanwhile, PV remains nearly unchanged for the silica-based series (≈12 cP), suggesting that the improvement is mainly due to enhanced yield stress rather than increased viscous drag.

**Table 7:** The rheological properties and filtration loss characteristics of fresh mud base. Mean ± SD (*n* = 3)^a^

Samples	*θ*_600_	*θ*_300_	PV (cP)	YP (lb per 100 ft^2^)	API fluid loss (mL per 30 min)	HPHT fluid loss (mL per 30 min)	Filter cake thickness (mm)	HPHT filter cake thickness (mm)
—	34 ± 1.73	22 ± 1.00	12 ± 1.00	10 ± 1.00	13 ± 1.32	30 ± 2.65	2.5 ± 0.30	2.25 ± 0.23
SiO_2_@melamine–chitosan (1000 ppm)	39 ± 1.00	27 ± 1.73	12 ± 1.73	15 ± 1.00	10 ± 1.32	24 ± 2.65	2.1 ± 0.26	1.89 ± 0.19
SiO_2_@melamine–chitosan (2000 ppm)	41 ± 2.00	29 ± 1.00	12 ± 1.00	17 ± 1.73	8.5 ± 1.73	19 ± 1.73	1.7 ± 0.17	1.53 ± 0.15
SiO_2_@melamine–chitosan (3000 ppm)	44 ± 1.73	32 ± 1.73	12 ± 2.00	20 ± 2.00	7 ± 1.73	16 ± 2.00	1.5 ± 0.17	1.35 ± 0.13
Fe_3_O_4_@melamine–chitosan (1000 ppm)	43 ± 1.00	30 ± 2.00	13 ± 1.73	17 ± 1.00	11 ± 1.80	25 ± 2.00	2.3 ± 0.26	2.07 ± 0.21
Fe_3_O_4_@SiO_2_@melamine–chitosan (2000 ppm)	48 ± 1.73	34 ± 1.00	14 ± 1.00	20 ± 1.73	9 ± 1.80	22 ± 1.73	2 ± 0.26	1.80 ± 0.18
Fe_3_O_4_@SiO_2_@melamine–chitosan (3000 ppm)	56 ± 1.00	38 ± 1.73	18 ± 1.73	20 ± 1.00	8.5 ± 0.87	20 ± 1.73	2 ± 0.20	1.80 ± 0.18

a Mud type: fresh water polymer mud, light (no barite), mud density: 9.1 ppg.

Filtration control is markedly improved with nanoparticle addition. API fluid loss decreases from 13 mL per 30 min (control) to 7 mL per 30 min at 3000 ppm for SiO_2_@melamine–chitosan, accompanied by a reduction in HPHT fluid loss from 30 to 16 mL per 30 min and a decrease in cake thickness from 2.5 to 1.5 mm. These trends are consistent with nanoparticle-assisted packing/bridging and cake densification, where nanosized particles occupy interstitial voids and reduce permeability pathways. Overall, the silica-based nanocomposite provides the strongest fluid-loss and cake-thickness reduction in this system, while the magnetic nanocomposite contributes more noticeably to rheological structuring.

### 3.3. The rheological properties and filtration loss characteristics of fresh mud base (12 ppg)

As summarized in Table 8, both nanocomposites improve the rheological profile of the fresh water-based weighted mud (12 ppg) and the effect strengthens with concentration. The control sample shows *θ*_600_/*θ*_300_ of 50/33, whereas values rise to 60/44 for SiO_2_@melamine–chitosan and 68/48 for Fe_3_O_4_@SiO_2_@melamine–chitosan at 3000 ppm. This progressive increase indicates stronger structure development, which can be rationalized by enhanced particle association promoted by melamine/chitosan-derived functionalities (adsorption and hydrogen bonding with clay and polymeric species). Yield point increases from 16 (control) to 28 at 3000 ppm for both additives, supporting improved suspension capacity, while PV remains relatively controlled (16–20 cP), implying no excessive flow resistance.

**Table 8:** The rheological properties and filtration loss characteristics of fresh mud base. Mean ± SD (*n* = 3)^a^

Samples	*θ*_600_	*θ*_300_	PV (cP)	YP (lb per 100 ft^2^)	API fluid loss (mL per 30 min)	HPHT fluid loss (mL per 30 min)	Filter cake thickness (mm)	HPHT filter cake thickness (mm)
—	50 ± 1.00	33 ± 1.00	17 ± 0.87	16 ± 1.00	13 ± 1.73	33 ± 6.24	3 ± 0.30	2.70 ± 0.27
SiO_2_@melamine–chitosan (1000 ppm)	54 ± 2.00	38 ± 1.00	16 ± 0.87	22 ± 1.73	11 ± 1.73	28 ± 4.58	2.6 ± 0.30	2.34 ± 0.24
SiO_2_@melamine–chitosan (2000 ppm)	56 ± 1.73	40 ± 1.00	16 ± 0.87	24 ± 1.00	10 ± 1.00	26 ± 4.00	2.3 ± 0.26	2.07 ± 0.21
SiO_2_@melamine–chitosan (3000 ppm)	60 ± 1.73	44 ± 1.73	16 ± 0.87	28 ± 2.00	8 ± 1.00	22 ± 3.61	2 ± 0.26	1.80 ± 0.19
Fe_3_O_4_@SiO_2_@melamine–chitosan (1000 ppm)	58 ± 1.73	41 ± 1.00	171.00	24 ± 2.00	12 ± 2.00	30 ± 5.29	2.9 ± 0.36	2.61 ± 0.26
Fe_3_O_4_@SiO_2_@melamine–chitosan (2000 ppm)	62 ± 1.73	44 ± 1.00	181.00	26 ± 1.73	10 ± 1.73	26 ± 4.58	2.6 ± 0.26	2.34 ± 0.24
Fe_3_O_4_@SiO_2_@melamine–chitosan (3000 ppm)	68 ± 2.00	48 ± 1.00	2028 ± 0.87	28 ± 2.65	9 ± 1.00	24 ± 4.36	2.5 ± 0.26	2.25 ± 0.23

a Mud type: fresh water polymer mud, weighted (12 ppg, barite).

Filtration results follow the same concentration-dependent improvement. Compared with the control (API/HPHT = 13/33 mL; cake thickness = 3.0 mm), both nanocomposites reduce filtrate volume and cake thickness with increasing concentration. The silica-based nanocomposite shows the largest filtration reduction at 3000 ppm (API/HPHT = 8/22 mL; cake thickness = 2.0 mm), consistent with nanoparticle-assisted packing that promotes a denser, less permeable cake. Overall, the magnetic additive provides a stronger rheology-building response, whereas the silica-based additive produces a more pronounced reduction in fluid loss and cake thickness.

### 3.4. The rheological properties and filtration loss characteristics of KCl-based water mud (9.1 ppg)

Table 9 shows that adding either nanocomposite to light KCl-based mud (9.1 ppg) enhances both rheological and filtration properties in a concentration-dependent manner. Compared with the control (*θ*_600_/*θ*_300_ = 28/18), *θ* readings increase steadily up to 40/28 for SiO_2_@melamine–chitosan and 48/32 for Fe_3_O_4_@SiO_2_@melamine–chitosan at 3000 ppm, indicating stronger structure development in the suspension. This behavior can be attributed to the melamine and chitosan network, which provides polar interaction sites capable of adsorption and hydrogen bonding with clay platelets and polymeric species, thereby improving particle connectivity. The increase in yield point (from 8 to 16 lb per 100 ft^2^) further supports a stronger low-shear network that improves carrying capacity. In contrast, PV changes only moderately (10–16 cP), indicating that the additives enhance the yield-stress component without disproportionately increasing viscous drag.

**Table 9:** The rheological properties and filtration loss characteristics of KCl-based water mud. Mean ± SD (*n* = 3)^a^

Samples	*θ*_600_	*θ*_300_	PV (cP)	YP (lb per 100 ft^2^)	API fluid loss (mL per 30 min)	HPHT fluid loss (mL per 30 min)	API filter cake thickness (mm)	HPHT filter cake thickness (mm)
—	28 ± 1.73	18 ± 1.73	10 ± 1.73	8 ± 1.00	15 ± 1.73	34 ± 6.08	3 ± 0.3	2.70 ± 0.27
SiO_2_@melamine–chitosan (1000 ppm)	34 ± 1.73	23 ± 1.73	11 ± 1.73	12 ± 1.00	12 ± 1.73	28 ± 4.58	2.4 ± 0.3	2.16 ± 0.22
SiO_2_@melamine–chitosan (2000 ppm)	37 ± 1.73	26 ± 1.73	11 ± 1.73	15 ± 2.00	10 ± 1.00	26 ± 4.00	2.3 ± 0.26	2.07 ± 0.21
SiO_2_@melamine–chitosan (3000 ppm)	40 ± 1.73	28 ± 1.73	12 ± 1.73	16 ± 1.00	9 ± 1.00	24 ± 4.58	2 ± 0.26	1.80 ± 0.19
Fe_3_O_4_@SiO_2_@melamine–chitosan (1000 ppm)	38 ± 1.73	25 ± 1.73	13 ± 1.73	12 ± 2.00	13 ± 2.00	30 ± 5.29	2.6 ± 0.26	2.34 ± 0.24
Fe_3_O_4_@SiO_2_@melamine–chitosan (2000 ppm)	42 ± 1.00	28 ± 1.00	14 ± 1.00	14 ± 1.73	11 ± 1.73	27 ± 4.58	2.5 ± 0.26	2.25 ± 0.23
Fe_3_O_4_@SiO_2_@melamine–chitosan (3000 ppm)	48 ± 1.73	32 ± 1.73	16 ± 1.73	16 ± 1.00	10 ± 1.00	25 ± 4.36	2.4 ± 0.26	2.16 ± 0.22

a Mud type: KCl polymer mud, light (3 wt% KCl, no barite), mud density: 9.1 ppg.

Consistent with the strengthened structure, filtration performance improves as nanoparticle loading increases. API fluid loss decreases from 15 mL per 30 min (control) to 9 mL per 30 min at 3000 ppm for the silica-based additive, while HPHT fluid loss decreases from 34 to 24 mL per 30 min and cake thickness drops from 3.0 to 2.0 mm. These trends are consistent with nanoparticle-assisted packing/bridging and improved cake densification, which reduce permeability and restrict fluid pathways. Overall, the magnetic nanocomposite tends to increase *θ* readings more strongly, whereas the silica-based system provides slightly greater cake densification and filtration reduction at higher concentrations.

### 3.5. The rheological properties and filtration loss characteristics of KCl-based water mud (12 ppg)

As shown in Table 10, both SiO_2_@melamine–chitosan and Fe_3_O_4_@SiO_2_@melamine–chitosan progressively strengthen the KCl-based weighted mud (12 ppg) as the concentration increases from 1000 to 3000 ppm. The steady rise in *θ*_600_/*θ*_300_ (from 50/33 in the control to 62/46 for the silica-based additive and 70/50 for the magnetic additive at 3000 ppm) indicates enhanced structure development in the suspension. This trend is consistent with stronger interparticle associations promoted by melamine/chitosan functionalities, which can provide multiple polar interaction sites (adsorption and hydrogen bonding) linking clay platelets, polymers, and the nanocomposite surface. Accordingly, yield point increases from 16 (control) to 30 at 3000 ppm for both nanocomposites, reflecting improved carrying/suspension capacity, while plastic viscosity changes only moderately (16–20 cP), suggesting that the additives reinforce the yield-stress component without inducing excessive flow resistance.

**Table 10:** The rheological properties and filtration loss characteristics of KCl-based water mud (12 ppg). Mean ± SD (*n* = 3)^a^

Samples	*θ*_600_	*θ*_300_	PV (cP)	YP (lb per 100 ft^2^)	API fluid loss (mL per 30 min)	HPHT fluid loss (mL per 30 min)	API filter cake thickness (mm)	HPHT filter cake thickness (mm)
—	50 ± 1.00	33 ± 1.00	17 ± 1.00	16 ± 1.00	15 ± 1.73	38 ± 7	3.5 ± 0.40	3.15 ± 0.32
SiO_2_@melamine–chitosan (1000 ppm)	56 ± 1.00	40 ± 1.00	16 ± 1.00	24 ± 1.00	12 ± 1.00	30 ± 5.57	2.9 ± 0.30	2.61 ± 0.26
SiO_2_@melamine–chitosan (2000 ppm)	58 ± 1.00	42 ± 2.00	16 ± 1.00	26 ± 1.00	11 ± 1.73	28 ± 5.00	2.6 ± 0.26	2.30 ± 0.23
SiO_2_@melamine–chitosan (3000 ppm)	62 ± 2.00	46 ± 1.00	16 ± 1.00	30 ± 2.00	9 ± 1.73	24 ± 4.58	2.3 ± 0.26	2.07 ± 0.21
Fe_3_O_4_@SiO_2_@melamine–chitosan (1000 ppm)	60 ± 1.00	43 ± 1.00	17 ± 2.00	26 ± 1.00	13 ± 1.00	32 ± 5.29	3.1 ± 0.36	2.79 ± 0.28
Fe_3_O_4_@SiO_2_@melamine–chitosan (2000 ppm)	64 ± 2.00	46 ± 1.00	18 ± 1.00	28 ± 1.00	11 ± 1.73	28 ± 5.29	2.8 ± 0.36	2.52 ± 0.25
Fe_3_O_4_@SiO_2_@melamine–chitosan (3000 ppm)	70 ± 1.00	50 ± 1.00	20 ± 1.00	30 ± 1.00	10 ± 1.73	26 ± 4.36	2.6 ± 0.26	2.34 ± 0.24

a Mud type: KCl polymer mud, weighted (3 wt% KCl, 12 ppg, barite).

Filtration behavior improves in parallel with the rheological enhancement. Relative to the control (API/HPHT = 15/38 mL; cake thickness = 3.5 mm), both nanocomposites reduce fluid loss and produce thinner cakes with increasing concentration. The strongest filtration improvement is obtained with SiO_2_@melamine–chitosan at 3000 ppm (API/HPHT = 9/24 mL; cake thickness = 2.3 mm), consistent with nanoparticle-assisted packing/bridging where nanosized particles occupy voids between larger solids, reduce permeability pathways, and promote a denser cake. Overall, Fe_3_O_4_@SiO_2_@melamine–chitosan provides a stronger rheology-building response, whereas SiO_2_@melamine–chitosan yields more pronounced fluid-loss and cake-thickness reduction.

### 3.6. Comparative analysis of SiO_2_@melamine–chitosan and Fe_3_O_4_@SiO_2_@melamine–chitosan in mud systems

**Fig. 9:**
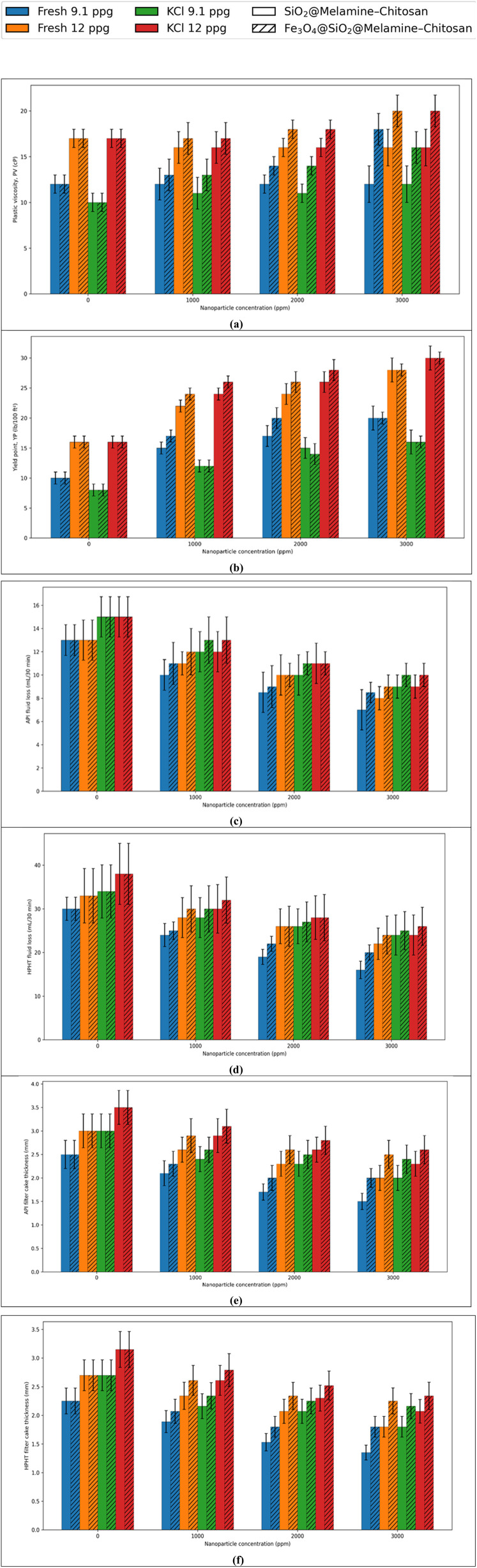
Effect of nanocomposite concentration (0–3000 ppm) on (a) PV, (b) YP, (c) API filtration volume, (d) HPHT filtration volume, (e) API filter-cake thickness, and (f) HPHT filter-cake thickness in fresh-water and KCl-based mud systems (9.1 and 12 ppg). Data are mean ± SD (*n* = 3).

For clarity, the results are discussed according to the mud system (fresh-water and KCl-based muds; 9.1 and 12 ppg), while the concentration-dependent trends for each measured parameter are presented in Fig. 9a–f (PV and YP in Fig. 9a and b; API FL and HPHT FL in Fig. 9c and d; API and HPHT cake thickness in Fig. 9e and f). All values are reported as mean ± SD (*n* = 3).

In fresh water-based light mud (9.1 ppg), Fe_3_O_4_@SiO_2_@melamine–chitosan increased PV from 12 to 18 cP (50%), while SiO_2_@melamine–chitosan kept PV nearly constant. However, the silica-based nanocomposite delivered the highest filtration-control performance, reducing API fluid loss from 13 to 7 mL per 30 min (46.15%) and HPHT fluid loss from 30 to 16 mL per 30 min (46.67%) at 3000 ppm. In fresh water-based weighted mud (12 ppg), SiO_2_@melamine–chitosan reduced API and HPHT fluid losses by 38.46% and 33.33%, respectively, while Fe_3_O_4_@SiO_2_@melamine–chitosan caused a greater PV increase. The same trend was observed in KCl-based muds. In the light KCl mud (9.1 ppg) and weighted KCl mud (12 ppg), SiO_2_@melamine–chitosan reduced API fluid loss from 15 to 9 mL per 30 min (40.00%) in both systems, whereas Fe_3_O_4_@SiO_2_@melamine–chitosan reduced it to 10 mL per 30 min and showed stronger viscosity enhancement.

Overall, the results reveal a consistent performance separation between the two nanocomposites. SiO_2_@melamine–chitosan is the more effective additive for filtration-loss reduction and cake-thickness control, while Fe_3_O_4_@SiO_2_@melamine–chitosan is more effective for rheological strengthening. The maximum filtration improvement was obtained in the fresh water-based light mud, where SiO_2_@melamine–chitosan achieved reductions of 46.15% in API fluid loss and 46.67% in HPHT fluid loss.

### 3.7. Mechanism of performance enhancement in water-based drilling fluids

The incorporation of Fe_3_O_4_@SiO_2_@melamine–chitosan and SiO_2_@melamine–chitosan into water-based drilling fluids resulted in measurable improvements in rheological parameters (particularly yield point) and significant reductions in API and HPHT fluid loss, accompanied by thinner filter cakes (Tables 7–10). These macroscopic changes constitute the primary experimental evidence obtained in this study. From a filtration standpoint, the consistent decrease in filtrate volume and cake thickness with increasing nanocomposite concentration suggests the formation of a less permeable filter cake structure. While direct microstructural characterization of the filter cake (*e.g.*, SEM imaging) was not performed, the observed trends are consistent with a nanoparticle-assisted packing or void filling effect. In this interpretation, nanoscale inorganic cores (Fe_3_O_4_ or SiO_2_) may occupy interstitial spaces between larger bentonite and barite particles during cake formation, thereby reducing permeability pathways. This explanation is supported indirectly by the progressive reduction in both API and HPHT filtration values as nanoparticle concentration increases. Regarding rheological behavior, the increase in yield point—without a proportionate rise in plastic viscosity—indicates reinforcement of the low-shear structural network rather than a general increase in viscous drag. This behavior suggests enhanced interparticle associations within the suspension. Given the presence of amino and hydroxyl groups in the melamine–chitosan network, specific interactions such as hydrogen bonding or surface adsorption onto clay platelets are plausible contributors. However, no direct adsorption measurements or *in situ* zeta potential analyses in the drilling fluid matrix were conducted in this study. Therefore, these interactions should be regarded as mechanistically reasonable but not directly verified under drilling conditions.

Zeta potential measurements performed in deionized water (*ζ* ≈ −1.8 to −2.1 mV) indicate near neutral surface charge for the nanocomposites under those conditions. This suggests that strong electrostatic stabilization is unlikely to be the dominant mechanism in aqueous dispersion. Consequently, steric effects arising from the organic shell and specific polar interactions are more likely contributors to the observed rheological modifications. Nevertheless, the electrochemical environment in drilling fluids—especially in the presence of KCl and bentonite—differs significantly from pure water, and further targeted studies would be required to quantify electrostatic contributions under realistic mud conditions.

### 3.8. Comparative discussion

The comparative result clearly illustrates the performance superiority of the triazine–chitosan network stabilized on silica and MNPs against other reported biodegradable additives in the literature. While additives such as okra powder, gum arabic, xanthan gum enhanced with diutan gum, carboxymethyl chitosan, and chitosan-*N*-(2-hydroxyl)-propyl trimethylammonium chloride generally improve rheological and fluid loss control properties, these improvements often require higher dosages (often in the wt% range) and do not consistently demonstrate the simultaneous reduction of both API and harsh HPHT (high temperature and high pressure) fluid loss alongside a thinner filter cake. The synthesized nanocomposite, achieving significant reduction in both fluid loss metrics (API and HPHT) and yielding the thinnest cake layer, proves a distinct performance and economic advantage at a ppm dosage, establishing this nanocomposite structure as an ideal solution for advanced drilling fluids (Table 11).^34,48–51^

**Table 11:** Comparative analysis of the present study *versus* previous literature

Additive	Typical dosage	Key reported outcomes
This work	1000–3000 ppm	Reduced standard fluid loss from 13 mL to 7 mL per 30 min; decreased HPHT fluid loss from 30 mL to 16 mL per 30 min; reduced filter cake thickness from 2.5 mm to 1.5 mm
Okra powder^48^	0.25–1 wt%	Enhanced fluid loss control and rheological properties
Gum arabic^49^	0.5–1.0 wt%	Reduced fluid loss; improved inhibition performance at ∼1 wt%
Xanthan gum enhanced with diutan gum^50^	0.02–0.05 lbm per gal	Effective fluid loss reduction contingent on temperature and aging conditions
Carboxymethyl chitosan^51^	0–2 wt%	Improved WBM rheology and filtration performance; medium molecular weight showed optimal inhibition
Chitosan-*N*-(2-hydroxyl)-propyl trimethylammonium chloride^34^	0.3–1.5 w/v%	Significant improvement in WBM performance (rheology, inhibition, and filtration)

## 4. Conclusion

In this study, two novel heterogeneous nanocomposites—Fe_3_O_4_@SiO_2_@melamine–chitosan and SiO_2_@melamine–chitosan—were successfully synthesized through the immobilization of a triazine-rich melamine–chitosan network onto silica-coated magnetite and silica nanoparticles. Comprehensive physicochemical characterization using FT-IR, XRD, FE-SEM, EDX, elemental mapping, and TGA confirmed the effective surface functionalization, uniform elemental distribution, and high thermal stability of the synthesized nanocomposites, indicating their suitability for demanding drilling environments.

The obtained results demonstrate that incorporating these nanocomposites into water-based drilling fluids can improve several key performance characteristics. In particular, the presence of the nanocomposites was associated with enhanced rheological behavior, evidenced by an increase in yield point that promotes effective solids suspension and improved carrying capacity, while maintaining acceptable viscosity levels. In addition, the systems containing the nanocomposites exhibited reduced API and high-pressure/high-temperature (HPHT) fluid loss were observed with increasing nanocomposite concentration, accompanied by the formation of more compact and less permeable filter cakes. In KCl-based systems, the partial suppression of clay hydration by K^+^ is well established. The additional reduction in filtration and increase in yield point observed after nanocomposite addition may indicate complementary effects, potentially involving surface coverage of clay platelets or modification of particle–particle contacts. However, without direct shale-swelling tests or adsorption isotherms, this interpretation remains inferential. Overall, the experimental data clearly demonstrate improved rheological stability and filtration control upon addition of the functionalized nanocomposites. The proposed mechanisms—nanoparticle-assisted packing, specific adsorption/hydrogen bonding, and steric network reinforcement—are consistent with the measured trends and existing literature but should be considered as plausible interpretations rather than directly proven processes. Further microstructural and interfacial characterization would be valuable in future work to confirm these hypotheses.

Notably, the magnetic nanocomposite (Fe_3_O_4_@SiO_2_@melamine–chitosan) consistently exhibited superior performance compared with its silica-based counterpart. This behavior is likely associated with the higher surface activity and adsorption affinity of iron-based nanoparticles, as well as their enhanced interaction with polymer chains and clay surfaces, which collectively contribute to improved rheological control and filtration performance. Such observations are consistent with recent reports highlighting the advantages of surface-functionalized magnetic nanoparticles in complex drilling fluid systems.

Overall, the findings suggest that combining biodegradable polymers with surface-engineered magnetic and silica nanoparticles represents a promising approach for improving drilling fluid performance. The resulting enhancements in rheological stability, filtration control, and thermal robustness indicate that these nanocomposites may serve as useful additives for advanced water-based drilling fluid formulations aimed at improving drilling efficiency and wellbore stability.

## Consent for publication

All authors agree to submit this manuscript to RSC Advances. They also confirm that this manuscript is their original work.

## Author contributions

Sara Motahari: doing laboratory work and preparing data, results analysis, writing, and editing. Ali Reza Kiasat: supervisor and presenter of research work, results analysis, writing, and editing.

## Conflicts of interest

The authors declare that they have no conflict of interest.

## Data Availability

The data that support the findings of this study are available from the corresponding author upon reasonable request.
